# Predicting Dementia in Cerebral Small Vessel Disease Using an Automatic Diffusion Tensor Image Segmentation Technique

**DOI:** 10.1161/STROKEAHA.119.025843

**Published:** 2019-09-12

**Authors:** Owen A. Williams, Eva A. Zeestraten, Philip Benjamin, Christian Lambert, Andrew J. Lawrence, Andrew D. Mackinnon, Robin G. Morris, Hugh S. Markus, Thomas R. Barrick, Rebecca A. Charlton

**Affiliations:** 1 From the Neurosciences Research Centre, Molecular and Clinical Sciences Research Institute, St George’s University of London, United Kingdom (O.A.W., E.A.Z., C.L., T.R.B.); 2 Department of Radiology, Charing Cross Hospital campus, Imperial College NHS Trust, United Kingdom (P.B.); 3 Wellcome Centre for Human Neuroimaging, UCL Queen Square Institute of Neurology, London, United Kingdom (C.L.); 4 Stroke Research Group, Clinical Neurosciences, University of Cambridge, United Kingdom (A.J.L., H.S.M.); 5 Atkinson Morley Regional Neuroscience Centre, St George’s NHS Healthcare Trust, London, United Kingdom (A.G.M.); 6 Department of Psychology, King’s College Institute of Psychiatry, Psychology, and Neuroscience, London, United Kingdom (R.G.M.); 7 Department of Psychology, Goldsmiths University of London, United Kingdom (R.A.C.).

**Keywords:** brain, cerebral small vessel disease, cerebrum, cognition, cognitive dysfunction, dementia, diffusion tensor imaging

## Abstract

Cerebral small vessel disease (SVD) is the most common cause of vascular cognitive impairment, with a significant proportion of cases going on to develop dementia. We explore the extent to which diffusion tensor image segmentation technique (DSEG; which characterizes microstructural damage across the cerebrum) predicts both degree of cognitive decline and conversion to dementia, and hence may provide a useful prognostic procedure.

**Methods—:**

Ninety-nine SVD patients (aged 43–89 years) underwent annual magnetic resonance imaging scanning (for 3 years) and cognitive assessment (for 5 years). DSEG-*θ* was used as a whole-cerebrum measure of SVD severity. Dementia diagnosis was based *Diagnostic and Statistical Manual of Mental Disorders V* criteria. Cox regression identified which DSEG measures and vascular risk factors were related to increased risk of dementia. Linear discriminant analysis was used to classify groups of stable versus subsequent dementia diagnosis individuals.

**Results—:**

DSEG-*θ* was significantly related to decline in executive function and global cognition (*P*<0.001). Eighteen (18.2%) patients converted to dementia. Baseline DSEG-*θ* predicted dementia with a balanced classification rate=75.95% and area under the receiver operating characteristic curve=0.839. The best classification model included baseline DSEG-*θ*, change in DSEG-*θ*, age, sex, and premorbid intelligence quotient (balanced classification rate of 79.65%; area under the receiver operating characteristic curve=0.903).

**Conclusions—:**

DSEG is a fully automatic technique that provides an accurate method for assessing brain microstructural damage in SVD from a single imaging modality (diffusion tensor imaging). DSEG-*θ* is an important tool in identifying SVD patients at increased risk of developing dementia and has potential as a clinical marker of SVD severity.

## Abstract 2

Supplemental Digital Content is available in the text.

Cerebral small vessel disease (SVD) is the primary cause of vascular cognitive impairment^1^ and vascular dementia.^2^ Clinically, patients with SVD present with lacunar strokes and are characterized by a decline in executive function (EF) and information processing speed (IPS), whereas memory functions appear to be relatively stable.^3^ Developing accurate biomarkers to track disease severity and identify individuals most at risk of converting to dementia is important to administer effective treatments and interventions.

Markers derived from magnetic resonance imaging (MRI) have been associated with cognitive decline in SVD. These include presence of white matter hyperintensities (WMH),^4^ gray matter (GM) atrophy,^5,6^ lacunar infarcts,^7^ cerebral microbleeds,^8,9^ and white matter (WM) microstructural damage detected using diffusion tensor imaging (DTI).^10–12^ WMH volume–derived and DTI-derived measures have also been shown to predict risk of receiving a dementia diagnosis in SVD.^13–16^

Markers of structural damage measured by MRI often co-occur in patients with SVD, and there is potential to combine multiple MRI markers into a unitary burden score. Such combined burden scores may provide a more accurate method for monitoring disease progress and establishing prognosis, with better predictions of cognitive decline than any single magnetic resonance marker.^17,18^ Additionally, an SVD burden score reduces the multiple comparisons required in statistical testing with multiple MRI methods. Unitary SVD burden scores have been generated by rating patients according to how many different MRI based markers they exhibit.^17,18^ For example, Huijts et al^17^ generated a scale of 0 to 4 based on the presence or absence of 4 SVD markers (WMH, lacunar infarcts, cerebral microbleeds, and perivascular spaces, assessed using 4 MRI acquisition protocols). They reported significant relationships between SVD burden score and EF, IPS, episodic memory (EM), and global cognition (GC). Associations between SVD burden scores and cognition have been replicated in more recent studies^19,20^; however, others have not found such relationships.^21^ Composite burden scores rely on considering continuous MRI data as binary (present/not present) constructs and may lose some statistical power and sensitivity in doing so. They also rely on data from multiple MRI acquisitions that often require manual segmentation and evaluation by experts.

This study assesses the application of an alternative SVD burden score derived from a fully automatic diffusion tensor image segmentation technique (DSEG).^22^ Although DTI is typically used to measure WM microstructure, it can be used to inform on the microstructure of all brain tissue, including GM and pathologically affected tissue.^22,23^ We have shown previously that it is possible to summarize information from DSEG into a single score (DSEG-*θ*) that describes the microstructure of the whole cerebrum.^24^ Furthermore, we found that change in DSEG-*θ* was related to change in conventional imaging markers of SVD, including WMH load, GM atrophy, lacunar infarcts, and cerebral microbleeds, in addition to DTI histogram parameters describing WM microstructure.^24^ As such, DSEG-*θ* is an automated technique that may provide a suitable biomarker of SVD severity based on a single imaging parameter (DTI), rather than relying on information from several different imaging modalities that often require manual segmentation.

Here, we test the hypothesis that baseline DSEG-*θ* scores and 3-year change in DSEG-*θ* obtained from a cohort of patients with SVD will be significantly related to decline in EF and IPS over a 5-year period. We also assess the hypothesis that differences in baseline DSEG-*θ* parameters are associated with an elevated risk of developing dementia over time. Lastly, we test the accuracy of DSEG-*θ* parameters in discriminating between individuals with SVD who go on to develop dementia and those who do not.

## Methods

### Participants

The data that support the findings of this study are available from Professor Markus upon reasonable request (hsm32@medschl.cam.ac.uk). Patients presenting with symptomatic SVD were recruited as part of the SCANS study (St George’s Cognition And Neuroimaging in Stroke).^12,25^ Inclusion criteria comprised of a clinical lacunar stroke syndrome^26^ with radiological evidence of an anatomically corresponding lacunar infarct ≤1.5 cm diameter. Further inclusion criteria required confluent regions of WMH as graded ≥2 on the modified Fazekas scale^27^ and fluency in English sufficient to enable cognitive testing. Cognitive assessments and MRI data were acquired at least 3 months after the last stroke to exclude acute effects on cognition. Exclusion criteria were contraindications to undergo MRI scanning, any cause of stroke other than SVD (eg, large artery stroke and cardioembolic stroke), current or history of central nervous system or major psychiatric disorder excluding migraine and depression, and any cause of WM disease other than SVD.

Patients were followed up annually with repeat MRI for 3 years and cognitive testing for 5 years. Patients were examined by a neurologist, and cardiovascular risk factors were recorded, including hypertension (systolic blood pressure >140 mm Hg or diastolic >90 mm Hg or treatment with antihypertensive drugs), hypercholesterolemia (serum total cholesterol >5.2 mmol/L or treatment with a statin), diabetes mellitus, and smoking status. Wandsworth (London) research ethics committee approved the study, and all patients provided written informed consent.

### Available SVD Data

At baseline, a total of 121 patients were recruited. MRI and neuropsychological data at multiple time points were available for 99 SVD patients (mean age, 68.42±9.98; range, 43–88; male=65). Of the 121 patients recruited, 103 attended >1 cognitive assessment. Eighteen patients only attended one assessment due to death (n=7), formal study withdrawal (n=6), house move (n=1), lost to follow-up (n=2), and withdrawal from full neuropsychological testing (n=2). Multiple MRI follow-up data were available for 99 of the remaining 103 participants. No participants were classified as demented at baseline.

### Magnetic Resonance Image Acquisition

Diffusion tensor images were acquired using a 1.5 Tesla GE Signa HDxt system (General Electric, Milwaukee, WI) with maximum gradient amplitude of 33 mT/m and a proprietary head coil. Acquisition matrix=96×96, field of view=240 mm×240 mm, echo time=93.4 ms, repetition time=1560 ms, 55 slices without any slice gaps to provide an isotropic voxel resolution of 2.5 mm x 2.5 mm x 2.5 mm. Diffusion-weighted spin-echo planar images were acquired with no diffusion weighting for 8 acquisitions (*b*=0 smm^-2^) followed by 25 noncollinear diffusion gradient directions and the negative of those diffusion gradient directions (*b*=1000 smm^-2^).

### DTI Analysis

#### Diffusion-Weighted Image Preprocessing

DTI preprocessing, including correction for eddy current distortions, and head movement have been described previously.^12^ Due to some participants not having full coverage of the cerebellum, it was removed from all scans using an automated technique.^24^

#### Diffusion Tensor Image Segmentation Technique

DSEG uses indices of isotropic (*p*) and anisotropic (*q*) diffusion.^28^ These measures may be visualized in a 2-dimensional Cartesian plane,^23^ the (*p,q*) space, in which it is possible to identify diffusion properties of GM, WM tissue, and cerebrospinal fluid, as well as pathologically affected tissue.^22–24^

DSEG is a fully automated DTI segmentation algorithm that separates (*p,q*) space into 16 discrete segments using a *k*-medians cluster analysis based on the magnitudes of the isotropic (*p*) and anisotropic (*q*) diffusion metrics for each voxel, given in mm2s^-1^.^22^ Each segment describes a unique diffusion profile representing tissue microstructural properties of each voxel assigned to that segment. This allows differences in the underlying isotropic and anisotropic diffusion characteristics to be determined for each individual across the entire cerebrum and compared between segments.

**Figure 1.**
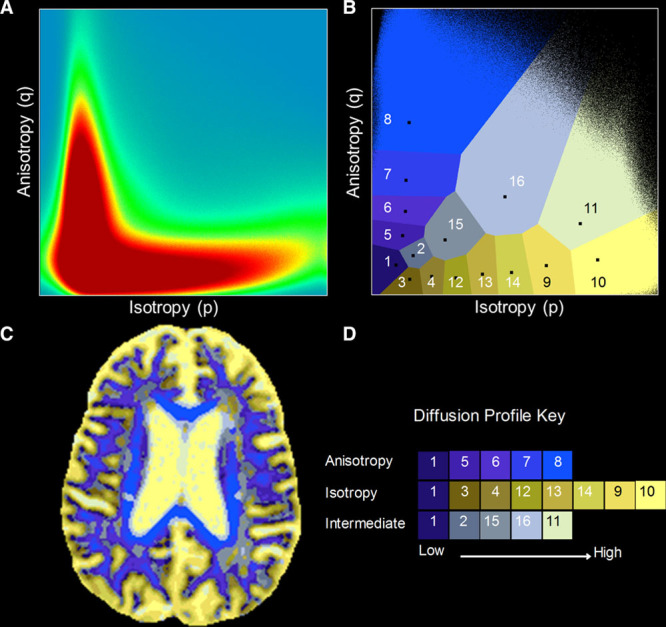
The diffusion tensor image segmentation technique (DSEG). **A**, The 2-dimensional histogram of *p* and *q* data from all voxels in the dataset. This represents the data that is used by DSEG to produce a whole-cerebrum segmentation using the diffusion tensor imaging (DTI) indices (*p* and *q*) to describe microstructural properties at each voxel. **B**, The resulting segmentation of the (*p,q*) plane represented in a Voronoi plot. Sixteen unique segments are generated around the segment centroids, which represent the median *p* and *q* values, shown as black squares. **C**, Illustrates how the segmentation in (*p*,*q*) space can be applied in any individual’s DTI native space. The patient shown is a 69-year-old who converted to dementia during the SCANS study (St George’s Cognition And Neuroimaging in Stroke). **D**, The diffusion profile key shows how each segment can be used to describe progressive levels of diffusion anisotropy and isotropy and intermediate levels of both.

Here, we perform DSEG simultaneously for all participants from the GENIE (St George’s Neuropsychology and Imaging in the Elderly study; healthy aging sample of 52 participants aged 53–91 years, 34 male)^29^ and SCANS (SVD)^7,10,12^ studies using *p* and *q* maps as described by Jones et al.^22^ DSEG performs a *k*-medians clustering of the probability density function (ie, 2-dimensional histogram, shown in Figure 1A) of *p* and *q*. A *k*-medians algorithm was used (as opposed to *k*-means) as the 2-dimensional histogram of *p* and *q* values were non-Gaussian thus cluster centroids were defined by the median. Full details of the technique have been described previously.^22,24^ The resulting segmentation of (*p,q*) space is represented in the Voronoi plot (Figure 1B).

#### DSEG Whole Brain Spectra

**Figure 2.**
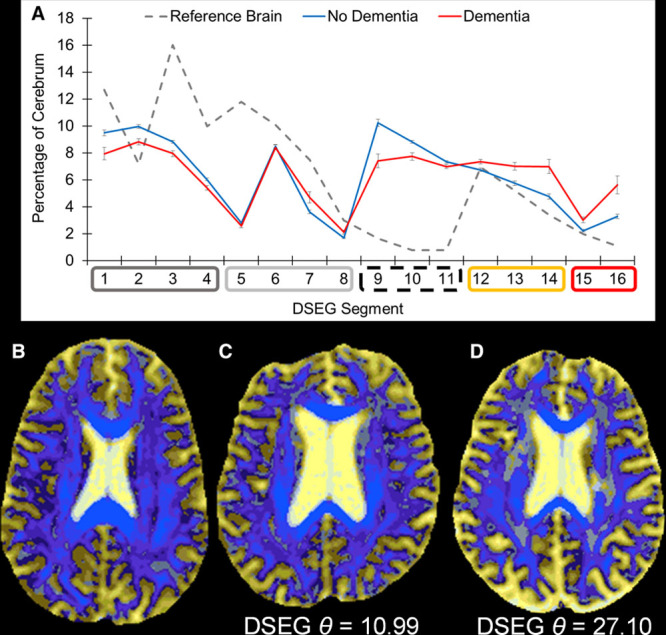
Diffusion tensor image segmentation technique (DSEG) spectra. **A**, DSEG spectra are generated by calculating the percentage of total cerebrum volume represented by each DSEG segment. Segments have been arranged in the colored boxes along the *x* axis to represent different tissue types: dark gray=gray matter (GM), pale gray=white matter (WM), dashed black=cerebrospinal fluid (CSF), orange=borderline tissue GM/CSF, and red=WM hyperintensity–related tissue damage. Here the reference brain DSEG spectrum is shown as the dashed gray line. The blue line represents the mean spectrum for all stable cerebral small vessel disease (SVD) patients, and the red line represents the mean spectrum for the dementia cohort. **B**, Axial DSEG image of the reference brain for calculating DSEG-*θ* (56-y-old). **C**, An axial DSEG image of a stable SVD patient who did not progress to dementia (66-y-old). **D**, An axial DSEG image of an SVD patient who did develop dementia during this study (69-y-old).

DSEG maps were generated for each individual; an example is shown in Figure 1C. To calculate DSEG spectra for each participant, the number of cerebrum voxels within each DSEG segment was determined, and the percentage contribution of each segment to the total cerebrum volume was calculated.^24^ This provides a subject-specific diffusion profile referred to as a DSEG spectrum (Figure 2A). This spectral information provides a signature diffusion profile containing information pertaining to GM and WM tissue, cerebrospinal fluid and includes regions with diffusion profiles that deviate from those of healthy tissue.

#### DSEG Summary Metric

**Figure 3.**
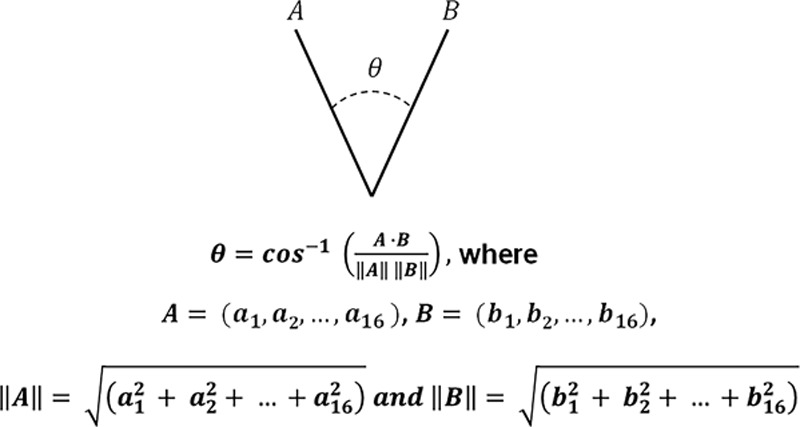
A schematic representation of the difference between vectors A and B. The equation shows how the dot product of 2 vectors is used to calculate *θ*. *θ* is similar to a correlation coefficient, and there is a lower angle when there is a higher positive correlation between vectors.

The angle, *θ*, between 2 vectors **A**=(a_1_, a_2_,…, a_16_) and **B**=(b_1_, b_2_,…, b_16_) may be given by the scalar product as shown in Equation 1 and provides a summary metric for the difference between 2 DSEG spectra that are represented by vectors A and B, as shown in Figure 3.

To ensure the metrics may be compared across participants, vector A was chosen to represent the DSEG spectrum representing the least damaged brain. This reference brain was identified using an iterative algorithm described previously.^24^ The reference brain selected by the algorithm corresponded to the DSEG spectrum of the youngest participant in the GENIE sample (aged 56 years). Vector B was then used to represent the DSEG spectra for each individual at each time point to calculate DSEG-*θ* for all individuals at each time point. The reference brain was selected in this way because on its own, the scalar product is nondirectional. By selecting a healthy brain free of SVD, we can impose direction on the angle *θ*, as a smaller angle reflects more similar total brain microstructural composition, whereas a larger *θ* will represent a greater divergence from healthy brain composition. It should be noted that the reference brain is used only as an anchor to generate DSEG-*θ* values. All statistical comparisons presented are based on within-subject change in DSEG-*θ* over time or group comparisons in DSEG-*θ* between participants who developed dementia and those who did not.

### Cognitive Assessment

A battery of standardized neuropsychological tasks was performed annually. Details of the full assessment have been published previously.^25^ EF was measured by the Trail Making Test, part B, a measure of phonemic fluency, and a modified Wisconsin Card-sorting Test. IPS was measured by the Digit Symbol Substitution Test, the BMIPB (The Brain Injury Rehabilitation Trust Memory and Information Processing Battery) Speed of Information Processing Speed Test, and the Grooved Pegboard Test. Working memory (WkM) was measured by the Wechsler Memory Scale-III digit forward and backward procedures. EM was measured by immediate and delayed recall from the Wechsler Memory Scale-III Logical Memory test and Visual Reproduction test. Individual measures were age-scaled using published normative data, converted to *Z* scores, and a mean composite score was calculated within each domain by averaging the *Z* scores in each domain (EF, IPS, WkM, EM, and GC comprising all measures). Premorbid intelligence was assessed using the National Adult Reading Test–restandardized, and the Mini-Mental State Exam was used as a dementia-screening tool.

### Conversion to Dementia

Information on conversion to dementia was available for all patients. Dementia was diagnosed using the *Diagnostic and Statistical Manual of Mental Disorders V*^30^ definition of “major neurocognitive disorder” and was present if individuals met one of the following criteria:

1. A diagnosis of dementia made in a memory clinic or equivalent clinical service.
2. After review of medical records and cognitive assessments by a neurologist and clinical neuropsychologist who were both blind to all MRI and risk factor information and who both agreed that *Diagnostic and Statistical Manual of Mental Disorders V* criteria were met.
3. A Mini-Mental State Exam score consistently <24, indicative of cognitive impairment^31^ and reduced capabilities in daily living as measured by a score ≤ 7 on the instrumental activities of daily living.^32^

Date of dementia onset was defined as the date of diagnosis in a clinical setting or the midpoint between testing sessions at which the diagnosis was established and the previous visit.

### Statistical Analysis

Linear mixed-effects models were applied using MLwiN^33^ to assess the effect of time on change in DSEG*-θ* over a 3-year period and cognition over a 5-year period. The intercept and slope of each participant’s linear trajectory were allowed to vary with both fixed and random effects. Fixed effect variation was accounted for by time, and random effect variation allowed for remaining interindividual differences. The average fixed effects slopes of time represent the average annualized change rate for a given measure. The Wald test was used to assess the goodness of fit for each model of change. Due to the discrepancy in testing periods for magnetic resonance data and cognitive data, the relationship between change in DSEG*-θ* and cognition were not explored using linear mixed-effects. Instead, the modeled gradients of change for each individual (in DSEG-*θ* and only the significantly declining cognitive domains) were analyzed using linear regression analysis in SPSS (V20.0). Models were adjusted for mean-centered baseline age, premorbid intelligence quotient, and sex.

#### Predicting Dementia

Student *t* tests and χ^2^ tests were used to assess differences in demographic characteristics, vascular risk factors, baseline DSEG-*θ*, and baseline cognition between stable patients and those that developed dementia.

Cox regression was applied to identify variables related to increased risk of conversion to dementia. Continuous variables were *Z* score transformed for ease of comparison. All variables that were significant in multivariable Cox regression were entered into a linear discriminant analysis.

Linear discriminant analysis was used as a classification technique to assess the sensitivity and specificity of markers in identifying individuals who converted to dementia. All linear discriminant analysis results reported represent output from leave-one-out cross-validation to reflect the stability of each model. The performance of each classification model was then assessed using sensitivity and specificity, the balanced classification rate (BCR),^34^ accuracy, and the area under the receiver operating characteristic curve (AUC), which is also known as the C statistic, and represents an overall indicator of model performance. In cases when there is a large difference in numbers between groups, the BCR may be considered a more useful definition of the overall classification performance.

## Results

### Cognitive Decline

Linear mixed-effects model results of change over time in DSEG-*θ* and cognitive scores are shown in Table 1. DSEG-*θ* increased significantly (*P*<0.001) over 3 years, indicating a progression of SVD burden. EF, IPS, and GC all decline significantly (*P*<0.001) over a 5-year period. There was no significant change in WkM (*P*<0.609) and EM (*P*<0.082). Baseline DSEG-*θ* and change in DSEG-*θ* were both related to decline in EF and GC (*P*<0.001) as shown in Table 2. There was no association with decline in IPS. As WkM and EM did not show significant change over time, their relationships with change in DSEG-θ were not investigated further.

**Table 1.**
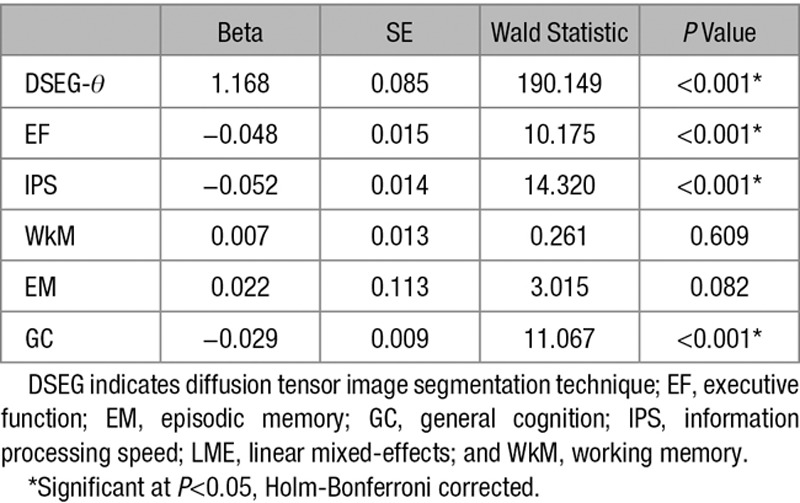
LME Models of Change in DSEG-*θ* (Over 3 Years) and Cognitive Domains (Over 5 Years)

**Table 2.**
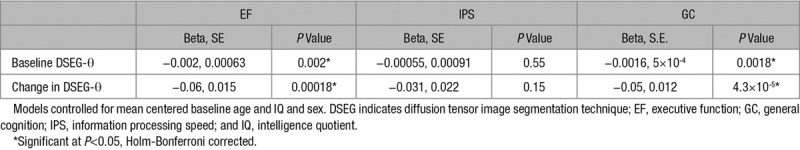
Linear Regression Showing the Relationships Between Baseline and Change in DSEG-θ With Decline in EF, IPS, and GC

### Predicting Dementia

Eighteen (18.2%) patients were identified as having converted to dementia. The mean time to dementia conversion was 3.31 years (1.40 SE).

Table I in the online-only Data Supplement shows the differences in SVD risk factors, cognitive scores, and DSEG-*θ* at baseline between individuals who went on to develop dementia and those who did not. There were no significant differences in demographic or vascular risk factors, including age, sex, and premorbid intelligence. However, there were significant differences in baseline DSEG-*θ*, EF, IPS, WkM, EM, and GC, as well as the Mini-Mental State Exam (*P*<0.001), with patients who developed dementia showing a higher level of overall brain damage and poorer cognitive functions at baseline. Although Mini-Mental State Exam scores were lower in the dementia group, they were still above the cutoff of 24 at baseline.

Univariate Cox regression of variables predicting risk of developing dementia revealed no significantly elevated risks associated with any demographic or vascular risk factors (Table II in the online-only Data Supplement). However, both DSEG-*θ* at baseline (hazard ratio, 3.331; 95% CI, 2.076–5.343) and change in DSEG-*θ* (hazard ratio, 3.905; 95% CI, 6.650) were associated with significant increases in risk of dementia (*P*<0.001). Mean DSEG spectra for demented and nondemented patients are shown in Figure 2A with example DSEG slices.

Table 3 shows the results of linear discriminant analysis for several classification models. All classification models, using either baseline DSEG-*θ* (BCR, 75.9 %; AUC, 0.839) or change in DSEG-*θ* (BCR, 81.50%; AUC, 0.881), were significant and identified individuals who went on to develop dementia and stable SVD patients. However, the most accurate model with a BCR, 79.65 and AUC, 0.903 is the model that includes DSEG-*θ* measures at baseline and follow-up in addition to age, sex, and premorbid intelligence.

**Table 3.**
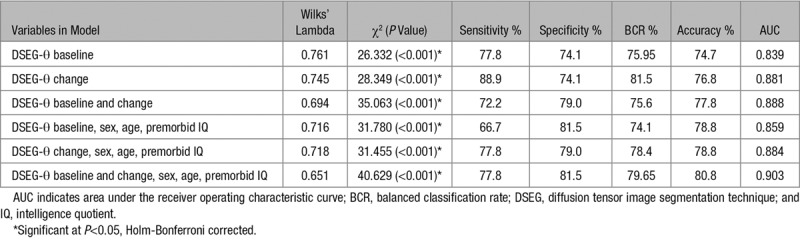
Discriminant Function Analysis Results for Predictive Models of Conversion to Dementia

## Discussion

This study has shown that DSEG-*θ* is sensitive to clinically important markers of SVD that can be used to accurately classify SVD patients. Results show that DSEG-*θ* predicts both decline in cognitive abilities and identifies those who are stable versus those who go on to develop dementia. Patients with greater SVD disease burden as measured by baseline DSEG-*θ* score and who had faster rates of disease progression as measured by change in DSEG-*θ* had faster rates of decline in EF and GC. These results show that DSEG-*θ* is useful as a preclinical marker for identifying individuals at risk of developing dementia in SVD and as a prognostic tool to measure the rate of disease progress and its impact on cognition.

Previous reports have shown that DTI techniques offer measures that are more sensitive to microstructural changes in WM in SVD than other common MRI metrics that are associated with cognitive decline and dementia.^11,13^ Here, we have shown that DSEG can be used as a marker of SVD that is related to decline in EF and GC. These associations are similar to those found between cognition and the SVD burden score,^17–20^ with the advantage that DSEG-*θ* is produced from a single DTI scan. Our results support the notion that DTI information can be organized to produce a single, whole-cerebrum marker of SVD severity related to cognitive decline even in patients who do not present with dementia. However, we did not find an association between DSEG-*θ* and IPS, this may be due to DSEG-*θ*, including information from the whole cerebrum and not just WM tracts. Information extracted specifically from the WM has been shown to be highly related to IPS.^10^

Furthermore, DSEG-*θ* also successfully predicts which individuals will go on to develop dementia. The performance of DSEG-*θ* in discriminant analysis is comparable to results presented by Barnes et al,^35^ who report an accuracy of 88% and an AUC of 0.810 for predicting dementia in older adults. Barnes et al^35^ used a late-life dementia risk index which included age, cognitive performance, body mass index, apoE ε4 alleles, WM disease (visual rating scale), ventricular enlargement, internal carotid artery thickening on ultrasound, history of bypass surgery, physical performance, and alcohol consumption. As such, the late-life dementia risk index is comprised of many different predictor variables based on multiple clinical assessments and MRI scan types. Although many of the measures used by Barnes et al^35^ are routinely collected, others require additional investigations and also additional scans. The approximate equal level of predictability using the single DTI scan that is required by DSEG compared with Barnes et al’s^35^ more complex model supports the clinical utility of the current technique.

Information from conventional MRI and DTI has been previously shown to predict dementia in the SCANS dataset.^16,36^ Using MD normalized peak height, WMH volume and premorbid intelligence predicted dementia with a BCR of 75.9% after leave-one-out cross-validation and an AUC of 0.85.^16^ Using baseline GM volumetric data conversion to dementia was predicted with a BCR of 74.4% and an AUC of 0.79.^36^ Our present findings suggest that application of DSEG can improve the BCR and AUC in classification models based on DTI information alone.

The finding that baseline DSEG-*θ* values predict dementia almost as well as change in DSEG-*θ* indicates that DSEG is sensitive to preclinical levels of SVD burden related to dementia. This is particularly important in the treatment of dementia for which any therapies are likely to be effective if applied before extensive damage has occurred.^37^ The accuracy of the discriminant analysis was improved when both baseline DSEG-*θ* and change in DSEG-*θ* were used. Therefore, DSEG may be used effectively in a clinical setting with a 2-tiered approach where initial assessment identifies individuals who should be monitored and change in DSEG-θ provides prognosis of disease progression.

A strength of the DSEG technique is that all analysis is performed in native DTI space and does not require additional preprocessing steps, such as coregistration, which is required when combining multiple imaging modalities, for tissue segmentation or voxel-wise statistical analysis of imaging data.^38^ Preprocessing steps required for combining different MRI methods inherently require interpolation of data and introduce error. Our study shows that DTI data can be used to generate a whole-cerebrum–based marker of SVD severity without the use of any other imaging modality or spatial averaging across individuals. Consequently, DSEG provides a fast and reliable alternative to conventional makers of SVD severity that may be used in a clinical setting without the use of advanced preprocessing.

A limitation of the technique is that the DSEG-*θ* metric is calculated by comparing each patient’s scan to that of a single healthy control. As such, the model of healthy aging in this study is a narrow representation of a healthy aging brain. However, with increasing availability of large biometric datasets (eg, the UK Biobank^39^), it will soon be possible to define normalized references of healthy aging brains and compare diseased individuals to an appropriate model of healthy aging. The MRI arm of the Biobank study aims to collect multimodal MRI (including DTI) for 100 000 participants (aged 40–69 years) by the year 2020. This would allow for representative samples of healthy aging individuals to be stratified by year or decade, defining a reference DSEG spectra potentially more relevant to each individual patient’s demographics. In addition, future studies will be required on larger data sets that include patients with evidence of SVD who do not have clinical lacunar stroke syndrome. This would allow for the assessment of the utility of DSEG in predicting cognitive decline and dementia in a larger clinical population compared with the stringent criteria used in the present study.

In conclusion, DSEG offers a highly accurate and sensitive marker of SVD severity in a single measure that can be used to distinguish between individuals who will and will not go on the develop dementia in a 5-year period. Furthermore, DSEG was highly related to SVD related cognitive decline, even in individuals who did not convert to dementia. Taken together, these findings suggest that DSEG may be used as a clinical tool to monitor SVD progression in patients and predict risk of developing dementia.

## Sources of Funding

This research was funded by UK Charity Research into Aging (Grant no. 374; Drs Williams and Barrick). The SCANS research study (St George’s Cognition And Neuroimaging in Stroke) was supported by a Wellcome Trust grant (081589). Recruitment was supported by the English National Institute of Health Research (NIHR) Clinical Stroke Research Network. This article was supported by grants from Alzheimer’s Research UK (ARUK-EXT2013-2 and ARUK-PG2016A-1; Drs Lawrence). Professor Markus is supported by an NIHR Senior Investigator award and the Cambridge University Hospital Comprehensive NIHR Biomedical Research Unit. The SCANS study was registered with the UK clinical research network (http://public.ukcrn.org.uk/, study ID:4577).

## Disclosures

None.

## Supplementary Material
