# Supplementary material for: Predicting Dementia in Cerebral Small Vessel Disease Using an Automatic Diffusion Tensor Image Segmentation Technique
Source: Stroke. 2019 Sep 12;50(10):2775–82. doi: 10.1161/STROKEAHA.119.025843 (PMC6756294; doi:10.1161/STROKEAHA.119.025843)
Supplement: Supplementary file 1 [file str-50-2775-s001.pdf]

SUPPLEMENTARY MATERIAL

Table I. Demographic, Vascular Risk Factor and Cognitive Characteristics of Non-dementing and Dementing SVD Patients

	Non-Dementia<br>n = 81	Dementia<br>n = 18	t/χ^2, p
**Baseline Age**	68.52 (1.083)	70.620 (2.620)	*t* = -0.81, *p* = .422
**Premorbid IQ**	101.20 (1.687)	94.89 (3.546)	*t* = 1.60, *p* = .114
**Women (%)**	29 (35.8)	4 (22.22)	χ^2 = 1.22, *p* = .269
**Diabetes (%)**	14 (17.28)	5 (27.80)	χ^2 = 1.05, *p* = .306
**Current smoker (%)**	15 (18.52)	6 (33.33)	χ^2 = 2.10, *p* = .350
**Ex-smoker (%)**	32 (39.51)	5 (27.80)	χ^2 = 2.10, *p* = .350
**Hypertension (%)**	75 (92.59)	17 (94.44)	χ^2 = 0.08, *p* = .782
**Mean diastolic BP**	82.55 (1.11)	79.39 (3.17)	*t* = 1.14, *p* = .258
**Mean systolic BP**	147.05 (2.44)	151.00 (4.80)	*t* = -0.703, *p* = .484
**Mean cholesterol**	4.43 (0.10)	4.12 (0.17)	*t* = 1.32, *p* = .191
**Mean BMI**	27.30 (0.55)	25.89 (1.49)	*t* = 1.04, *p* = .300
**Baseline DSEG θ**	18.26 (0.80)	28.68 (1.78)	*t* = -5.52, *p* <.001*
**Baseline EF**	-0.67 (1.00)	-1.83 (0.90)	*t* = 5.08, *p* < .001*
**Baseline IPS**	-0.75 (0.83)	-1.95 (0.42)	*t* = 9.89, *p* < .001*
**Baseline WkM**	-0.050 (0.88)	-0.54 (0.98)	*t* = 6.07, *p* < .001*
**Baseline EM**	0.24 (0.89)	-0.92 (0.82)	*t* = 2.09, *p* =.039*
**Baseline GC**	-0.46 (0.68)	-1.41 (0.58)	*t* = 6.36, *p* < .001*
**Baseline MMSE**	28.21 (1.75)	24.82 (3.93)	t = 4.04, *p* < .001*

***significant difference at p < .05, Holm-Bonferroni corrected.**

Table II: Univariable Cox Regression showing Vascular Risk Factors and DSEG *θ* Predicting Risk of Developing Dementia

	**Hazard Ratio**	**95 % CI**	***p***
**Baseline Age**	1.217	0.738 – 2.006	.443
**Premorbid IQ**	0.681	0.417 - 1.112	.124
**Gender**	0.518	0.170 - 1.575	.246
**Diabetes**	1.232	0.44 – 3.46	.692
**Current smoker**	1.910	0.64 – 5.71	.246
**Ex-smoker**	0.767	0.24 – 2.42	.650
**Mean diastolic BP**	0.651	0.383 – 1.107	.113
**Mean systolic BP**	1.383	0.879 – 2.178	.162
**Mean cholesterol**	0.757	0.512 – 1.122	.165
**Mean BMI**	0.790	0.472 – 1.323	.370
Baseline* DSEG *θ	3.331	2.076 – 5.343	< .001*
Change in* DSEG *θ	3.905	2.293 – 6.650	< .001*

* Significant at p = .05
